# Spillover of Azithromycin Mass Drug Administration and Child Survival: A Secondary Analysis of a Cluster-Randomized Clinical Trial

**DOI:** 10.1001/jamanetworkopen.2025.19693

**Published:** 2025-07-10

**Authors:** Ahmed M. Arzika, Abdou Amza, Ramatou Maliki, Bawa Aichatou, Ismael Mamane Bello, Diallo Beidi, Nasser Galo, Nasser Harouna, Alio M. Karamba, Sani Mahamadou, Moustapha Abarchi, Almou Ibrahim, Carolyn Brandt, Elodie Lebas, Brittany Peterson, Zijun Liu, Catherine E. Oldenburg, Thuy Doan, Travis C. Porco, Benjamin F. Arnold, Thomas M. Lietman, Kieran S. O’Brien

**Affiliations:** 1 Centre de Recherche et Interventions en Santé Publique, Birni N’Gaoure, Niger; 2 Programme Nationale de Santé Oculaire, Niamey, Niger; 3 Francis I. Proctor Foundation, University of California, San Francisco; 4 Department of Ophthalmology, University of California, San Francisco; 5 Department of Epidemiology and Biostatistics, University of California, San Francisco; 6 Institute for Global Health Sciences, University of California, San Francisco

## Abstract

**Importance:**

World Health Organization guidelines on azithromycin mass drug administration for child survival target infants aged 1 to 11 months, although prior studies included those aged 1 to 59 months. The AVENIR trial suggested that infants aged 1 to 11 months have lower mortality if children aged 12 to 59 months in the same household are also included.

**Objective:**

To assess the possibility of a spillover effect by examining the association of azithromycin and mortality among children aged 1 to 11 months in subgroups defined by the presence of a child aged 12 to 59 months in the same household.

**Design, Setting, and Participants:**

This exploratory secondary analysis of the AVENIR (Azithromycine Pour la Vie des Enfants au Niger: Implementation et Recherche) adaptive cluster-randomized clinical trial was performed in 3000 rural and periurban communities in Niger. AVENIR communities were randomized to 3 arms and followed up for 2 years (November 24, 2020, to July 31, 2023). Study arms consisted of children aged 1 to 59 months receiving azithromycin (child arm); infants aged 1 to 11 months receiving azithromycin with placebo to children aged 12 to 59 months (infant arm); and children aged 1 to 59 months receiving placebo (placebo arm). Participants, investigators, data collectors, and data analysts were masked to randomization.

**Intervention:**

A single 20-mg/kg dose of oral azithromycin or placebo administered by study staff biannually.

**Main Outcomes and Measures:**

All-cause mortality in infants aged 1 to 11 months (deaths per 1000 person-years) measured through biannual census. Subgroups were defined by the presence of a child aged 12 to 59 months in the household recorded during the census.

**Results:**

After exclusions, 2883 communities and 98 969 infants aged 1 to 11 months were included in the analysis. Among the 23 770 infants in allocation 1 at baseline, mean (SD) age was 6.2 (3.1) months and 11 974 (50.4%) were female. Mortality was 18.5 (95% CI, 16.7-20.4) deaths per 1000 person-years in the child arm, 22.3 (95% CI, 20.0-24.7) in the infant arm, and 23.9 (95% CI, 21.6-26.2) in the placebo arm. The incidence rate ratio comparing mortality in the child and infant arms among children with an older sibling was 0.78 (95% CI, 0.65-0.93) compared with 0.91 (95% CI, 0.73-1.15; *P* = .26 for interaction) among those without. Comparing the infant and placebo arms, the incidence rate ratio among children with an older sibling was 0.96 (95% CI, 0.81-1.14) compared with 0.90 (95% CI, 0.71-1.12; *P* = .61 for interaction) among those without.

**Conclusions and Relevance:**

In this secondary analysis of a cluster-randomized clinical trial, interaction for the presence of a older sibling was not statistically significant, but results were consistent with lower mortality among infants aged 1 to 11 months living with older, treated children.

**Trial Registration:**

ClinicalTrials.gov Identifier: NCT04224987

## Key Points

**Question:**

Does the association between azithromycin mass administration and mortality in children 1 to 11 months of age differ by whether an older azithromycin-treated child was present in the same household?

**Findings:**

In a secondary analysis of a cluster-randomized clinical trial including 98 969 infants, communities distributing azithromycin to all children aged 1 to 59 months saw lower mortality among those aged 1 to 11 months if those children had an older treated sibling, although the interaction was not statistically significant. Among communities distributing azithromycin only to infants aged 1 to 11 months, mortality was the same in this age group regardless of whether an older untreated sibling was in the household.

**Meaning:**

Although underpowered, these results were consistent with a spillover effect of azithromycin treatment among younger children when older siblings are treated.

## Teaser

This secondary analysis of a cluster-randomized clinical trial tests the association of azithromycin mass administration with mortality among infants aged 1 to 11 months in subgroups defined by the presence or absence of an older child in the same household.

## Introduction

The MORDOR (Macrolides Oraux Pour Réduire les Décès Avec un Oeil sur la Résistance) cluster-randomized clinical trial demonstrated that biannual mass drug administration (MDA) with azithromycin to children aged 1 to 59 months reduced all-cause mortality by 14% in Malawi, Niger, and Tanzania.^1^ Subgroup analyses suggested the potential for stronger effects in younger children, although the trial was not powered to detect this interaction.^1^ Based on these findings, the World Health Organization released conditional guidelines on this intervention, recommending that azithromycin MDA be considered in high-mortality settings with eligibility limited to infants aged 1 to 11 months.^2^ This age group was selected given high mortality and possible stronger effects in the youngest children, along with concern about increasing antimicrobial resistance.^2,3,4,5,6^

The AVENIR (Azithromycine Pour la Vie des Enfants au Niger: Implementation et Recherche) response-adaptive cluster-randomized clinical trial aimed to compare the impact of targeting azithromycin MDA to different ages.^7,8^ This 3-arm trial randomized communities in Niger to receive azithromycin for children aged 1 to 59 months (child arm), azithromycin to infants aged 1 to 11 months and placebo to children aged 12 to 59 months (child arm), or placebo to infants and children aged 1 to 59 months (placebo arm) during a 2-year period.^8^ Communities receiving azithromycin MDA for infants and children aged 1 to 59 months had 14% (95% CI, 7%-22%) lower mortality compared with communities receiving placebo, whereas communities receiving azithromycin MDA for infants aged 1 to 11 months had a nonsignificant 6% (95% CI, −8% to 19%) decrease in mortality compared with communities receiving placebo.^8^ When comparing the 2 azithromycin arms, the trial demonstrated that azithromycin administered to infants and children aged 1 to 59 months reduced mortality in the group aged 1 to 11 months by 17% (95% CI, 4%-28%) compared with treating those aged 1 to 11 months alone.^8^ This finding has important implications for countries considering this intervention, suggesting that the youngest children benefit the most when older children are also treated. Given the burden of mortality from infectious diseases among the youngest children,^9^ spillover effects are plausible.

In this post hoc subgroup analysis of the AVENIR trial, we aimed to further explore this spillover effect. We examined the association of azithromycin MDA with mortality among infants aged 1 to 11 months in subgroups defined by the presence or absence of an older child in the same household. The study design and analysis were conducted at the cluster level to reflect the community-based nature of the intervention and its mechanism, which likely involve a reduction in community transmission of pathogens responsible for mortality in children younger than 5 years. We hypothesized that azithromycin MDA would be associated with a stronger reduction in mortality in infants aged 1 to 11 months who lived in a household where children aged 12 to 59 months also received azithromycin.

## Methods

### Design and Oversight

AVENIR was designed to compare all-cause mortality after 2 years in communities targeting different age groups with azithromycin MDA in a cluster-randomized, response-adaptive clinical trial in Niger. Communities were randomized to 1 of 3 arms: (1) biannual azithromycin administration to infants and children aged 1 to 59 months (child arm); (2) biannual azithromycin administration to infants aged 1 to 11 months and placebo administration to children aged 12 to 59 months (infant arm); or (3) biannual administration of placebo to infants and children aged 1 to 59 months (placebo arm). Detailed methods have been reported previously, and the trial protocol is included in Supplement 1.^7,8^ This exploratory subgroup analysis assessed the possibility of a spillover effect by examining the association between azithromycin MDA and mortality in infants in subgroups defined by the presence of an older child in the household.

The Comité National Éthique pour la Recherche en Santé in Niger and the Institutional Review Board at the University of California, San Francisco, provided ethical approval. A data and safety monitoring committee was empaneled before the study began. The data and safety monitoring committee oversaw participant safety and study progress by reviewing quarterly progress reports and convening annual meetings. Verbal informed consent was obtained from community leaders before study activities began. Verbal consent was also obtained from heads of household for census activities and from caregivers of eligible children for treatment administration. Written informed consent was obtained from caregivers for children aged 30 to 42 days, given the potential risk of macrolide-associated infantile hypertrophic pyloric stenosis.^10^ This report follows the Consolidated Standards of Reporting Trials (CONSORT) guideline for cluster-randomized clinical trials.

### Setting, Participants, and Eligibility

Accessible rural and periurban communities with populations ranging from 250 to 2499 (median, 619 [IQR, 356-822]) people in the Dosso and Tahoua regions were eligible for inclusion based on data available from the last national census conducted in 2012. Eligibility for treatment included children who were 1 to 59 months of age at the current census, weighed at least 3 kg, and had no known allergy to macrolides. Eligibility for inclusion in this subgroup analysis included communities with treatment and mortality data on infants aged 1 to 11 months at the beginning of an intercensus interval.

### Randomization and Masking

The *grappe* was used as the randomization unit in AVENIR and will be referred to as *community*. One unmasked biostatistician (T.C.P.) generated the response-adaptive randomization sequence in R, version 4.5 (R Foundation for Statistical Computing). The adaptive allocation has been described in depth previously.^8^ Briefly, the trial began with communities randomized with equal probability (1 of 3) to each of the arms. Two further allocations randomized newly enrolled communities after 12 and 18 months. Probabilities were estimated using negative binomial regression with count of deaths as the outcome, an offset for person-time at risk, and indicators for the 3 arms using all outcome measurements available at each adaptation. Based on the probability that each arm had the lowest mortality rate in infants and children aged 1 to 59 months, newly enrolled communities had a higher probability of being allocated to the arm with the lowest mortality rate. Once a community was randomized, it retained its assigned treatment. Masking was achieved through use of matching azithromycin and placebo, which were prepared to be identical in appearance, smell, and packaging. Investigators, participants, treatment administrators, outcome assessors, and half of the biostatistics team remained masked during the trial. As described in the Results section, several postrandomization exclusions occurred largely due to outdated census information before data were collected.

### Census

Every 6 months, a trained study team visited every household in the study area to conduct a census, deliver treatment, and monitor the vital status of included children. For each household, the team recorded demographic data for children aged 1 to 59 months using a mobile application (CommCare; Dimagi). Subsequent census data collection included vital status of previously included children (alive, died, moved, or unknown) and entry of new children and households. The subgroup of interest in this study was defined by the presence or absence of a child aged 12 to 59 months in the household (referred to hereafter as a sibling) using the data collected during the census, with sibling status determined at the beginning of each intercensus interval.

### Intervention

At each census, azithromycin and placebo were administered as a single oral suspension dose of 20 mg/kg (Pfizer Inc), and treatment administration was recorded in the mobile application. Dose was determined by weight using a hanging scale (ADE M111600; GmbH & Co) for children aged 1 to 59 months. Dose was determined with the height-based dosing pole used in Niger for children aged 12 to 59 months.^11^ Caregivers were instructed to report adverse events that occurred within 28 days after MDA to the study team.

### Outcome

The outcome of interest in this exploratory (nonprespecified) subgroup analysis is the community-level all-cause mortality rate (deaths per 1000 person-years at risk) in infants aged 1 to 11 months after 2 years of distributions. A death was counted if a child was present on one census and absent on the subsequent census due to death. Person-time at risk was calculated as time alive and eligible for treatment while living in the study area, with children who died contributing half the person-time for the last intercensus interval. Children who moved or had unknown status contributed no person-time to the interval in question.

Two pairwise comparisons were examined. The first compared mortality among infants aged 1 to 11 months in both azithromycin arms (child and infant arms) by subgroup. In this comparison, all infants aged 1 to 11 months received azithromycin, and children aged 12 to 59 months were treated with azithromycin in the child arm and with placebo in the infant arm. The second compared mortality among infants aged 1 to 11 months in the infant and placebo arms by subgroup. For this comparison, children aged 12 to 59 months received placebo in both arms whereas infants aged 1 to 11 months received azithromycin only in the infant arm, essentially serving as a negative control.^12^

### Sample Size

The sample size for this analysis was fixed by the original trial design,^8^ which anticipated that inclusion of 8525 intercensus intervals and 1116 communities per arm would provide 80% power to detect a 10% relative reduction in mortality among children aged 1 to 59 months and 19% relative reduction in mortality among infants aged 1 to 11 months at α = .05. This assumed a baseline mortality of 27 deaths per 1000 person-years in participants aged 1 to 59 months (SD, 0.023) and 45 deaths per 1000 person-years in infants aged 1 to 11 months (SD, 0.074) based on prior data.^1^

### Statistical Analysis

We aimed to examine effect modification by sibling subgroup for each pairwise comparison of interest on both the multiplicative and additive scales. All analyses were based on intention to treat and conducted at the community level. Poisson regression was used to estimate incidence rate ratios (IRRs) and assess effect modification on the multiplicative scale, including the count of deaths as the outcome and community person-time at risk as an offset, with observations for each community’s intercensus interval. Terms were included for treatment arm, sibling subgroup, treatment × subgroup interaction, and the randomization allocation to account for the adaptive design. Permutation *P* values for the interactions were estimated for the F statistic from the Wald test, with a robust variance-covariance matrix to compare models with and without the interaction term, clustered at the community level. To estimate incidence rate differences (IRDs) and effect modification on the additive scale, we used a Poisson model and G-computation approach. The fitted model was used to estimate the number of deaths under each counterfactual treatment and subgroup for each community. Mortality incidence rates and their differences were calculated using the estimated number of deaths and observed person-time at risk in each community under each treatment arm and subgroup status. Additive interaction contrasts were calculated to assess interaction on the additive scale, and permutation tests were used to estimate *P* values.^13^ To estimate the proportion of the overall association of azithromycin with mortality among infants aged 1 to 11 months due to targeting these infants vs including children aged 12 to 59 months, we used a similar Poisson regression model with count of deaths as the outcome, community person-time as the offset, and terms for the adaptive allocation along with terms for whether a community’s treatment allocation included infants aged 1 to 11 months or children aged 12 to 59 months. Proportion due to each treatment approach was estimated as the percentage reduction in mortality with each treatment approach divided by the overall percentage reduction in mortality with azithromycin among infants aged 1 to 11 months. For all analyses, we estimated 95% CIs using bootstrap resampling (10 000 replicates) by resampling communities with replacement. Interaction 2-sided *P*  = .05 was considered statistically significant. R, version 4.5, was used for all analyses.

## Results

**Figure 1.**
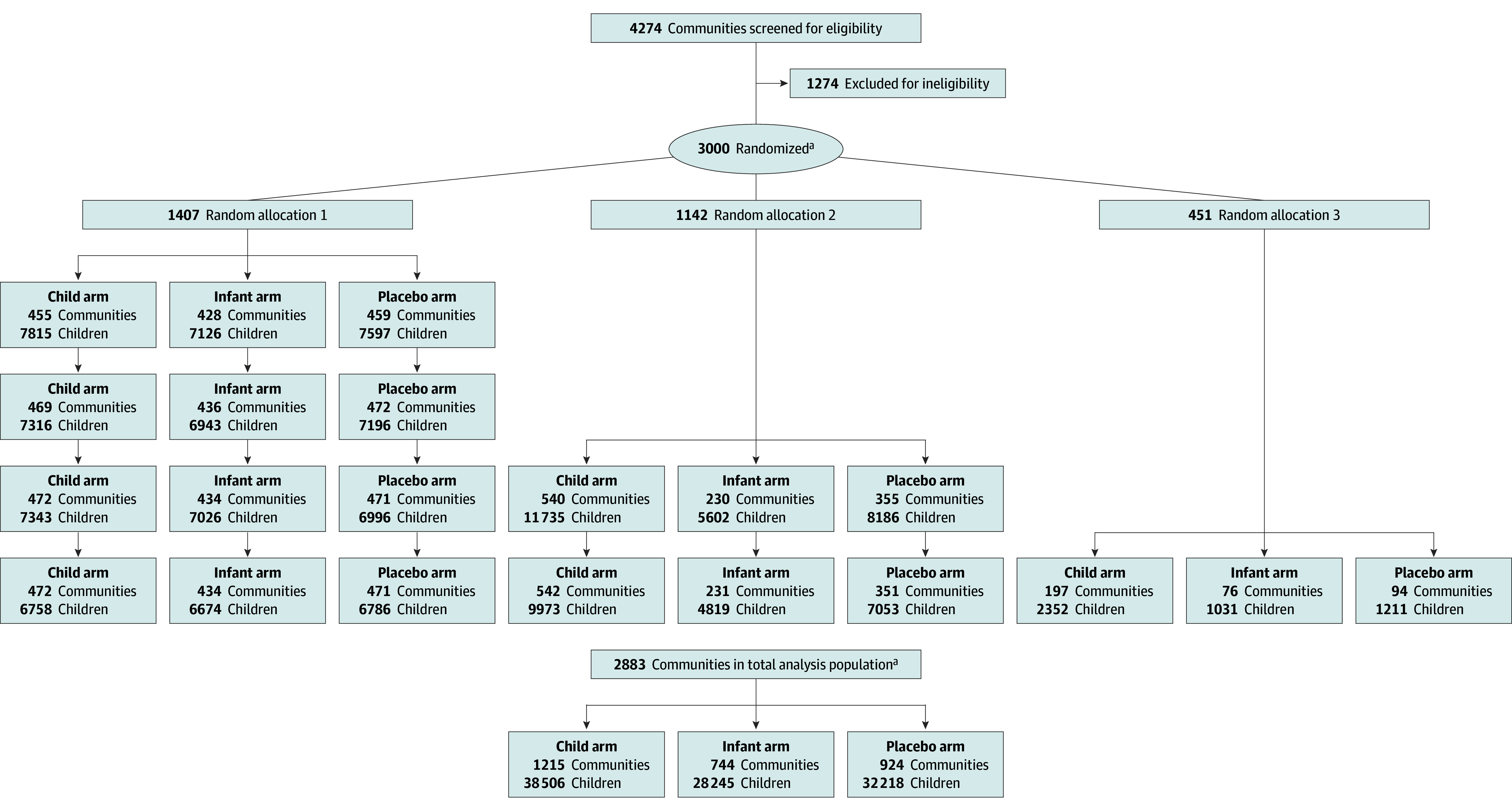
Participant Flow Diagram Communities were randomized to 1 of 3 arms: biannual azithromycin administration to infants and children aged 1 to 59 months (child arm); biannual azithromycin administration to infants aged 1 to 11 months and placebo administration to children aged 12 to 59 months (infant arm); or biannual administration of placebo to infants and children aged 1 to 59 months (placebo arm). The number of communities is shown for each component of the flow diagram. The total number of communities included in the main trial is shown in the allocation section, whereas for the follow-up section only eligible communities contributing to analyses by census interval and arm are shown. The number of children aged 1 to 11 months is also shown by allocation, census interval (1-4), and arm. As the trial used a dynamic cohort design, communities and children were able to enter, leave, and re-enter the trial in the different intercensus intervals. ^a^We excluded 117 communities after randomization, including 39 with census inaccuracies, 7 refusals, 40 in insecure areas, 2 moved communities, 2 protocol deviations, 1 with only 1 census household, and 26 with no person-time for infants. Only unique children are counted and displayed in the total analysis population.

Of the 4274 communities originally screened for eligibility, 3000 were enrolled and randomized. After exclusions related to security challenges, outdated national census information, and child age, 2883 communities and 98 969 unique infants aged 1 to 11 months were included in the analysis. Among the 23 770 infants included at baseline, mean (SD) age was 6.2 (3.1) months, 11 974 (50.4%) were female, and 11 796 (49.6%) were male (Figure 1 and Table). The trial began enrollment November 24, 2020, included 5 MDAs approximately every 6 months, and completed follow-up July 31, 2023. Baseline characteristics were similar by arm and across the 3 random allocations (Table). Mean (SD) treatment coverage across MDAs was 97.1% (5.5%) (eTable 1 in Supplement 2). No communities were lost to follow-up.

**Table.** Baseline Characteristics Among Participants

Characteristic	Treatment arm^a^			Overall
	Child	Infant	Placebo	
**Random allocation 1**				
No. of communities	463	431	463	1357
Infants aged 1-11 mo, No./total No. (%)	8220/44 412 (18.5)	7569/41 064 (18.4)	7981/43 702 (18.3)	23 770/129 178 (18.4)
No. of infants aged 1-11 mo per community, mean (SD)	17.75 (13.72)	17.56 (14.30)	17.24 (12.44)	17.52 (13.48)
Infant age, mean (SD), mo	6.18 (3.05)	6.18 (3.08)	6.11 (3.11)	6.16 (3.08)
Infant sex, No. (%)				
Female	4128 (50.2)	3824 (50.5)	4022 (50.4)	11 974 (50.4)
Male	4092 (49.8)	3745 (49.5)	3959 (49.6)	11 796 (49.6)
Sibling aged 12-59 mo in household, No. (%)				
Yes	4638 (59.0)	4319 (59.7)	4617 (60.6)	13 574 (59.7)
No	3224 (41.0)	2915 (40.3)	3005 (39.4)	9144 (40.3)
**Random allocation 2**				
No. of communities	543	231	355	1129
Infants aged 1-11 mo, No./total No. (%)	12 061/68 924 (17.5)	5731/32 926 (17.4)	8428/48 763 (17.3)	26 220/150 613 (17.4)
No. of infants aged 1-11 mo per community, mean (SD)	22.21 (16.65)	24.81 (18.46)	23.74 (18.48)	23.22 (17.63)
Infant age, mean (SD), mo	5.77 (3.03)	5.73 (3.01)	5.72 (3.03)	5.75 (3.03)
Infant sex, No. (%)				
Female	6069 (50.3)	2879 (50.2)	4245 (50.4)	13 193 (50.3)
Male	5992 (49.7)	2852 (49.8)	4183 (49.6)	13 027 (49.7)
Sibling aged 12-59 mo in household, No. (%)				
Yes	6851 (59.6)	3155 (57.7)	4726 (59.0)	14 732 (59.0)
No	4641 (40.4)	2316 (42.2)	3290 (41.0)	10 247 (41.0)
**Random allocation 3**				
No. of communities	209	82	99	390
Infants aged 1-11 mo, No./total No. (%)	2490/12 674 (19.6)	1116/5320 (21.0)	1272/6209 (20.5)	4878/24 203 (20.2)
No. of infants aged 1-11 mo per community, mean (SD)	11.91 (12.23)	13.61 (14.99)	12.85 (11.06)	12.51 (12.57)
Infant age, mean (SD), mo	6.07 (2.93)	5.92 (2.97)	6.18 (2.94)	6.07 (2.95)
Infant sex, No. (%)				
Female	1221 (49.0)	536 (48.0)	620 (48.7)	2377 (48.7)
Male	1269 (51.0)	580 (52.0)	652 (51.3)	2501 (51.3)
Sibling aged 12-59 mo in household, No. (%)				
Yes	1261 (52.8)	574 (53.5)	664 (55.5)	2499 (53.6)
No	1129 (47.2)	499 (46.5)	532 (44.5)	2160 (46.4)

^a^ Treatment arms include biannual azithromycin administration to infants and children aged 1 to 59 months (child arm); biannual azithromycin administration to infants aged 1 to 11 months and placebo administration to children aged 12 to 59 months (infant arm); or biannual administration of placebo to infants and children aged 1 to 59 months (placebo arm). Cluster-level characteristics are summarized for the 3 treatment arms and 3 random allocations, which occurred at months 0, 12, and 18. Communities contributing to analyses that had measurements included in the respective allocation’s first census are included.

**Figure 2.**
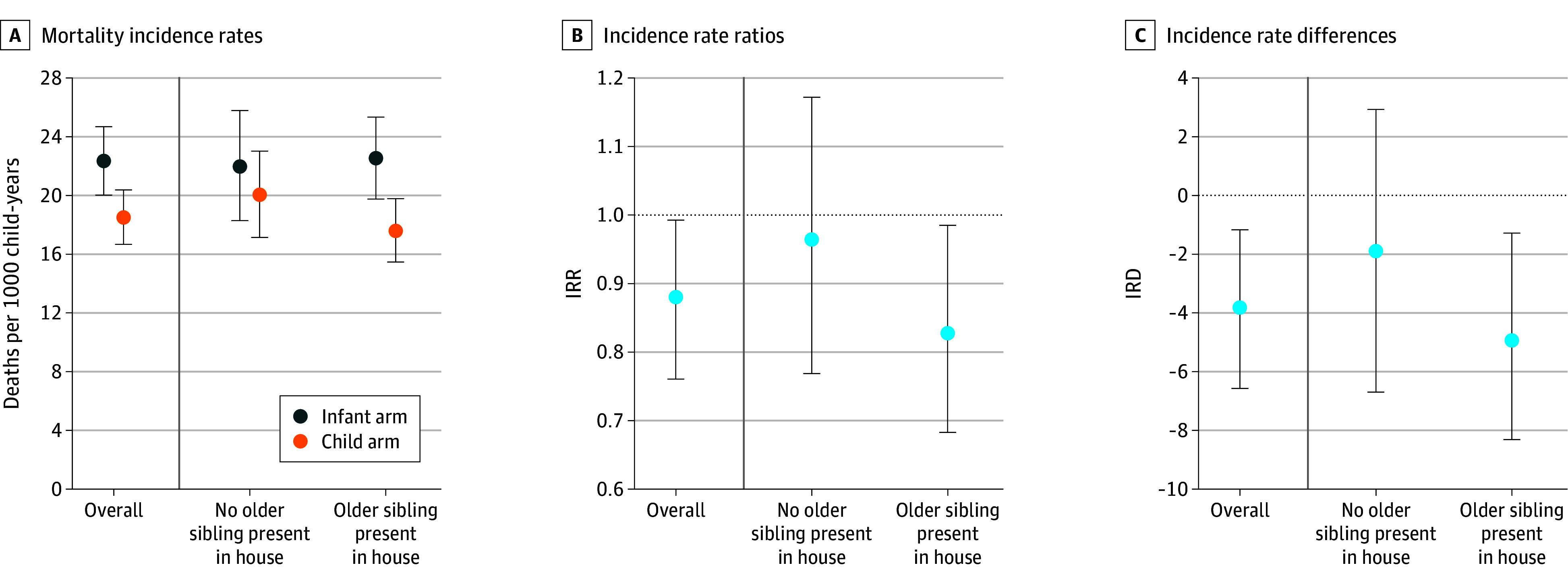
Comparison of Child vs Infant Arms Data are shown for the child arm, with biannual azithromycin administration to infants and children aged 1 to 59 months, and the infant arm, with biannual azithromycin administration to infants aged 1 to 11 months and placebo administration to children aged 12 to 59 months. Mortality incidence rates, incidence rate ratios (IRRs), and incidence rate differences (IRDs) are compared among infants aged 1 to 11 months by treatment arm and presence of an older sibling (12-59 months of age) in the household. IRRs comparing mortality rate by arm and presence of a sibling were estimated using Poisson regression accounting for the adaptation and clustering. IRDs comparing mortality by arm and presence of an older sibling were estimated using G-computation based on the Poisson model. Supportive data are found in eTable 2 in Supplement 2.

**Figure 3.**
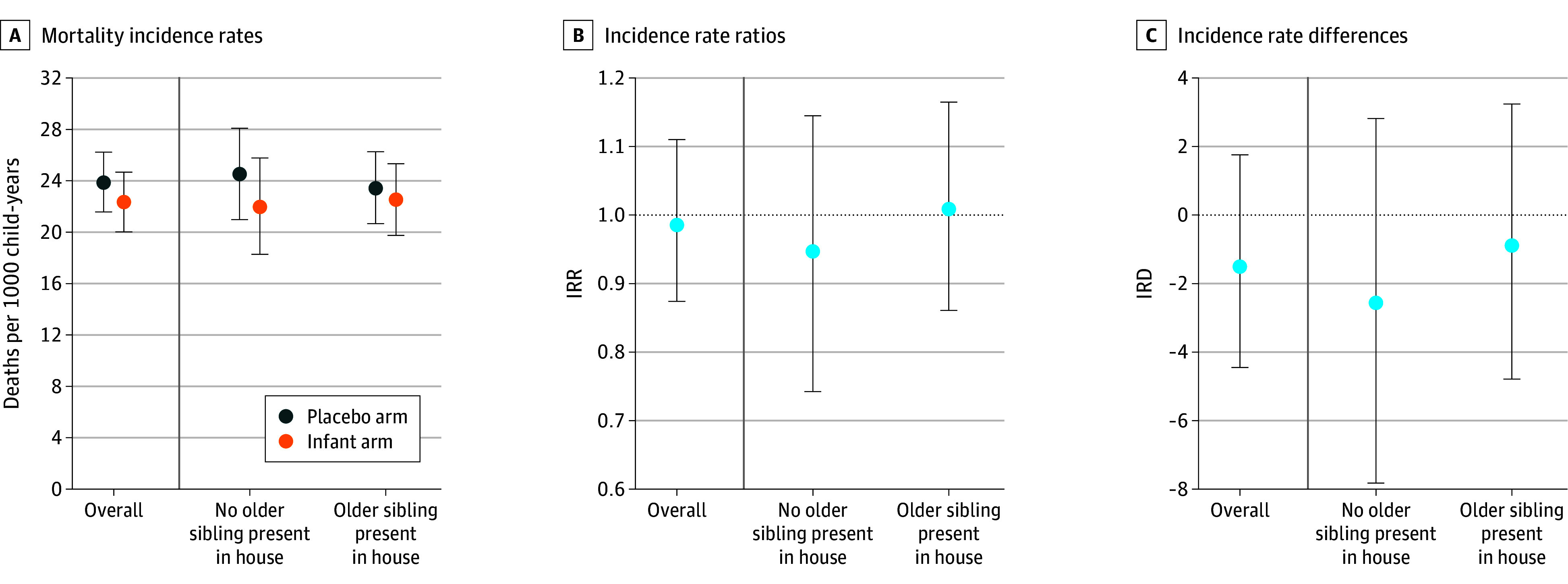
Comparison of Infant vs Placebo Arms Data are shown for the placebo arm, with biannual administration of placebo to infants and children aged 1 to 59 months, and the infant arm, with biannual azithromycin administration to infants aged 1 to 11 months and placebo administration to children aged 12 to 59 months. Mortality incidence rates, incidence rate ratios (IRRs), and incidence rate differences (IRDs) are compared among infants aged 1 to 11 months by treatment arm and presence of an older sibling (12-59 months of age) in the household. IRRs comparing mortality rate by arm and presence of a sibling were estimated using Poisson regression accounting for the adaptation and clustering. IRDs comparing mortality by arm and presence of an older sibling were estimated using G-computation based on the Poisson model. Supportive data are found in eTable 2 in Supplement 2.

Among infants aged 1 to 11 months, 1432 deaths and 69 590 person-years of observation were included in analyses. The incidence rates of mortality among these infants were 18.5 (95% CI, 16.7-20.4) deaths per 1000 person-years at risk in the child arm, 22.3 (95% CI, 20.0-24.7) deaths per 1000 person-years in the infant arm, and 23.9 (95% CI, 21.6-26.2) deaths per 1000 person-years in the placebo arm (Figure 2, Figure 3, and eTable 2 in Supplement 2). The overall reduction in mortality at 1 to 11 months of age in communities receiving any azithromycin compared with placebo was 23% (95% CI, 11%-33%), with 23.5% (95% CI, 1.2%-72.7%) explained by treating infants with azithromycin and 76.5% (95% CI, 27.3%-98.8%) explained by also treating children 12 to 59 months of age with azithromycin.

When comparing the child and the infant arms, the IRR among infants with an older sibling in the household was 0.78 (95% CI, 0.65-0.93), whereas the IRR among infants without an older sibling was 0.91 (95% CI, 0.73-1.15) (Figure 2 and eTable 3 in Supplement 2). The multiplicative interaction contrast was 0.92 (95% CI, 0.71-1.18; *P* = .26). On the additive scale, the IRD comparing mortality among infants aged 1 to 11 months in the child and infant arms for those with an older sibling was −4.94 (95% CI, −8.31 to −1.28). Among infants without an older sibling, the IRD was −1.90 (95% CI, −6.70 to 2.93) (Figure 2 and eTable 3 in Supplement 2). The additive interaction contrast was −1.39 (95% CI, −7.21 to 4.53; *P* = .31).

When comparing the infant and the placebo arms, the IRR among infants with an older sibling in the household was 0.96 (95% CI, 0.81-1.14). The IRR among infants without an older sibling was 0.90 (95% CI, 0.71-1.12) (Figure 3 and eTable 3 in Supplement 2). The multiplicative interaction contrast was 1.07 (95% CI, 0.83-1.40; *P* = .61). The IRD comparing mortality in the infant and placebo arms among infants with an older sibling was −0.90 (95% CI, −4.79 to 3.24). Among infants without an older sibling, the IRD was −2.56 (95% CI, −7.82 to 2.82) (Figure 3 and eTable 3 in Supplement 2). The additive interaction contrast was 1.66 (95% CI, −4.53 to 8.18; *P* = .36).

Five serious adverse events were reported during the trial. Two of these occurred among infants aged 1 to 11 months (eTable 4 in Supplement 2).

## Discussion

The AVENIR trial suggested that azithromycin MDA to infants and children aged 1 to 59 months acts in part through a herd effect involving younger children receiving protection from older treated children. In this analysis, we aimed to further explore this spillover effect in subgroups defined by the presence of an older child in the household. Overall, we were unable to detect statistically significant evidence of an effect modification in sibling subgroups on the multiplicative or additive scales in any set of comparisons. This is not unexpected, given that mortality is a rare event, especially among a small subset of the population such as infants aged 1 to 11 months, so power to test for interaction was lower than in the primary analysis. Nevertheless, mortality among infants aged 1 to 11 months was lowest when infants lived in a household with a child aged 12 to 59 months who was treated with azithromycin, and we estimated that 76.5% of the mortality reduction among infants was due to treatment of the older children. When no older children were treated, observed mortality reductions were smaller if older untreated children were present in the household vs absent, suggesting that the presence of older untreated children reduced the overall effect. All of these results are consistent with the theorized spillover effect.

The mechanism of a spillover effect of treating children aged 12 to 59 months with azithromycin on mortality in infants aged 1 to 11 months presumably involves a reduction in within-household transmission of infectious diseases associated with mortality in that age group, given the close proximity expected among household contacts. Studies of azithromycin MDA^9,14,15,16,17,18,19,20,21,22^ have identified reductions in morbidity and mortality from respiratory infections, diarrhea, and malaria, which are known to be important causes of mortality in both infants and all children younger than 5 years in this setting. The strongest evidence for spillover effects in other settings has been identified among interventions that act via reduced transmission of disease, including vaccines and MDA.^23^ For example, studies of azithromycin MDA for trachoma^24,25^ have identified reductions in the prevalence of ocular chlamydial infection in untreated groups.

Several studies have examined spillover effects of azithromycin on untreated groups, including both positive and negative effects. One secondary analysis of the MORDOR cluster-randomized clinical trial^26^ suggested the potential for a mortality benefit of azithromycin MDA to infants and children aged 1 to 59 months among untreated children of the same age in the same community, although the analysis was underpowered. A few studies^27,28,29^ have also focused on the potential for negative spillover effects of this intervention on antimicrobial resistance and intestinal microbiome diversity in untreated groups. One of these studies^27^ was another secondary analysis of the MORDOR trial that similarly suggested the potential for a small increase in macrolide resistance determinants in the nasopharynx of untreated children in communities receiving azithromycin MDA, but this study was underpowered and was unable to demonstrate a statistically significant effect. At the same time, one small household-randomized study^28,29^ did not find evidence of increases in macrolide resistance determinants or in intestinal microbiome diversity in untreated children living in the same household as a child treated with azithromycin. Given that the impact of MDA programs relies on community-level effects in addition to individual-level effects, household- or individual-level studies may not be ideal for identifying community-level spillover effects.

### Strengths and Limitations

Strengths of this study include the randomized placebo-controlled design, which limits concerns over bias in estimation of intervention effects. In addition, inclusion of 3 arms focused on different age groups with use of placebo allowed for examination of effects in age subgroups that was not possible in previous trials. Twice yearly census data collection by experienced field teams enabled close monitoring of child mortality, reducing the potential for measurement error. AVENIR also used a population-based approach to sampling, including all households in eligible communities across most of 2 regions, reducing the potential for selection bias and ensuring generalizability of these results to similar settings in Niger and other Sahelian countries.

An important limitation of this design is statistical power. Even in high-mortality settings, mortality is a rare event, and most subgroup analyses are underpowered. The large simple trial design also required simple and streamlined data collection for a trial of this size to be feasible to implement.^30^ We were thus unable to include an evaluation of cause-specific mortality or infectious disease incidence, which may have allowed for a more nuanced analysis focused on specific infectious causes of death most likely to be associated with a spillover effect involving azithromycin MDA.

## Conclusions

In this secondary analysis of a cluster-randomized clinical trial, although we were underpowered to detect statistically significant evidence of a spillover effect of azithromycin MDA on mortality in infants aged 1 to 11 months in subgroup analyses, our findings are consistent with an association between treating older children and mortality in younger children. The main trial results support implementation of azithromycin MDA to infants and children aged 1 to 59 months to achieve the greatest benefit. The analyses presented herein further support this conclusion and suggest that the spillover mechanism may include treating older siblings.

## Notes

Supplement 1.
Trial Protocol

Supplement 2. **eTable 1**. Treatment Coverage by Treatment Arm and Census **eTable 2**. Mortality Rates, Incidence Rate Ratios (IRRs), and Incidence Rate Differences (IRDs) Among Infants Aged 1 to 11 Months by Treatment Arm and Presence of an Older Sibling (12-59 Months) in Household **eTable 3**. Multiplicative and Additive Interaction Contrasts **eTable 4.** Serious Adverse Events by Treatment Arm

Supplement 3.
Data Sharing Statement
