# Supplementary material for: Spillover of Azithromycin Mass Drug Administration and Child Survival: A Secondary Analysis of a Cluster-Randomized Clinical Trial
Source: JAMA Netw Open. 2025 Jul 10;8(7):e2519693. doi: 10.1001/jamanetworkopen.2025.19693 (PMC12246876; doi:10.1001/jamanetworkopen.2025.19693)
Supplement: Supplement 2. — eTable 1. Treatment Coverage by Treatment Arm and Census eTable 2. Mortality Rates, Incidence Rate Ratios (IRRs), and Incidence Rate Differences (IRDs) Among Infants Aged 1 to 11 Months by Treatment Arm and Presence of an Older Sibling (12-59 Months) in Household eTable 3. Multiplicative and Additive Interaction Contrasts eTable 4. Serious Adverse Events by Treatment Arm [file jamanetwopen-e2519693-s002.pdf]

Supplementary Online Content

Arzika AM, Amza A, Maliki R, et al. Potential spillover association of azithromycin mass drug administration with child survival: a secondary analysis of a cluster-randomized clinical trial. *JAMA Netw Open.* 2025;8(7):e2519693. doi:10.1001/jamanetworkopen.2025.19693

eTable 1. Treatment Coverage by Treatment Arm and Census
eTable 2. Mortality Rates, Incidence Rate Ratios (IRRs), and Incidence Rate Differences (IRDs) Among Infants Aged 1 to 11 Months by Treatment Arm and Presence of an Older Sibling (Aged 12-59 Months) in Household
eTable 3. Multiplicative and Additive Interaction Contrasts
eTable 4. Serious Adverse Events by Treatment Arm

This supplementary material has been provided by the authors to give readers additional information about their work.

© 2025 Arzika AM et al. *JAMA Network Open*.

eTable 1. Treatment Coverage by Treatment Arm and Census

Census Month	Child Azithromycin 1-59m Mean, SD [Min, Max]	Infant Azithromycin 1-11m Mean, SD [Min, Max]	Placebo Mean, SD [Min, Max]
**0**	96.2%, 11.2% [0.0%, 100.0%]	97.5%, 4.5% [71.1%, 100.0%]	97.0%, 8.0% [0.0%, 100.0%]
**6**	96.3%, 6.4% [43.6%, 100.0%]	96.6%, 5.0% [63.0%, 100.0%]	96.5%, 5.4% [54.3%, 100.0%]
**12**	98.5%, 3.2% [76.7%, 100.0%]	98.1%, 3.5% [80.8%, 100.0%]	98.3%, 3.4% [76.6%, 100.0%]
**18**	98.4%, 4.3% [0.0%, 100.0%]	97.9%, 3.9% [80.3%, 100.0%]	97.9%, 4.0% [80.3%, 100.0%]
**24**	94.5%, 17.8% [0.0%, 100.0%]	92.5%, 20.9% [0.0%, 100.0%]	93.8%, 18.9% [0.0%, 100.0%]
**Overall**	97.4%, 5.8% [0.0%, 100.0%]	96.8%, 5.2% [70.7%, 100.0%]	97.0%, 5.4% [25.9%, 100.0%]

Treatment coverage is calculated as the number of eligible children 1-59 months old treated with azithromycin or placebo divided by the number of eligible children 1-59 months censused at each census.
Max, maximum; Min, minimum; m, month; SD, standard deviation

© 2025 Arzika AM et al. *JAMA Network Open*.

eTable 2. Mortality Rates, Incidence Rate Ratios (IRRs), and Incidence Rate Differences (IRDs) Among Infants Aged 1 to 11 Months by Treatment Arm and Presence of an Older Sibling (Aged 12-59 Months) in Household

2a. Child azithromycin 1-59m vs infant azithromycin 1-11m

Subgroup	Child Azithromycin 1-59m Mortality Rate (95% CI)	Infant Azithromycin 1-11m Mortality Rate (95% CI)	Incidence rate ratio (95% CI)	Incidence rate difference (95% CI)
**Overall**	18.5 (16.7 to 20.4)	22.3 (20.0 to 24.7)	0.83 (0.72, 0.96)	-3.82 (-6.57, -1.17)
**Older sibling (12-59 months) in household**	17.6 (15.5 to 19.8)	22.5 (19.8 to 25.3)	0.78 (0.65, 0.93)	-4.94 (-8.31, -1.28)
**No older sibling (12-59 months) in household**	20.0 (17.1 to 23.0)	22.0 (18.3 to 25.8)	0.91 (0.73,1.15)	-1.90 (-6.70, 2.93)

2b. Infant azithromycin 1-11m vs placebo

Subgroup	Infant Azithromycin 1-11m Mortality Rate (95% CI)	Placebo Mortality Rate (95% CI)	Incidence rate ratio (95% CI)	Incidence rate difference (95% CI)
**Overall**	22.3 (20.0 to 24.7)	23.9 (21.6 to 26.2)	0.94 (0.81, 1.08)	-1.51 (-4.45, 1.76)
**Older sibling (12-59 months) in household**	22.5 (19.8 to 25.3)	23.4 (20.7 to 26.3)	0.96 (0.81, 1.14)	-0.90 (-4.79, 3.24)
**No older sibling (12-59 months) in household**	22.0 (18.3 to 25.8)	24.5 (21.0 to 28.1)	0.90 (0.71, 1.12)	-2.56 (-7.82, 2.82)

IRRs and IRDs are shown for the each pairwise comparison overall and by the presence of an older sibling (12-59 months old) in the household.

© 2025 Arzika AM et al. *JAMA Network Open*.

eTable 3. Multiplicative and Additive Interaction Contrasts

Comparison	Multiplicative Interaction Contrast (95% CI)	*P*-value	Additive Interaction Contrast (95% CI)	*P*-value
Child Azithromycin 1-59 vs Placebo	0.85 (0.65 to 1.12)	0.26	-3.04 (-8.57 to 2.54)	0.31
Infant Azithromycin 1-11 vs Placebo	1.07 (0.82 to 1.40)	0.61	1.66 (-4.53 to 8.18)	0.36

Multiplicative interaction was evaluated with an interaction term for treatment and subgroup using Poisson regression accounting for the adaptation and clustering. Additive interaction was evaluated using g-computation based on the Poisson model. *P*-values were estimated using permutation tests.

© 2025 Arzika AM et al. *JAMA Network Open*.

eTable 4. Serious Adverse Events by Treatment Arm

Treatment Arm	Treatment Date	Number of days between treatment and event	Age in months when event occurred	Description
Placebo 1-59m	2021-01-25	0	11	Hospitalization for malaria
Placebo 1-59m	2021-12-13	0	22	Hospitalization for malaria
Placebo 1-59m	2022-05-10	0	34	Hospitalization for vomiting, diarrhea, and fever
Infant Azithromycin 1-11m	2022-05-06	0	36	Malaria, fever, diarrhea, vomiting
Child Azithromycin 1-59m	2023-05-12	2	3	Diarrhea, fever, and vomiting

© 2025 Arzika AM et al. *JAMA Network Open*.
