# Supplementary material for: Spillover of Azithromycin Mass Drug Administration and Child Survival: A Secondary Analysis of a Cluster-Randomized Clinical Trial
Source: JAMA Netw Open. 2025 Jul 10;8(7):e2519693. doi: 10.1001/jamanetworkopen.2025.19693 (PMC12246876; doi:10.1001/jamanetworkopen.2025.19693)
Supplement: Supplement 3. — Data Sharing Statement [file jamanetwopen-e2519693-s003.pdf]

Data Sharing Statement

Arzika. Potential Spillover Association of Azithromycin Mass Drug Administration With Child Survival. *JAMA Netw Open*. Published July 10, 2025.
doi:10.1001/jamanetworkopen.2025.19693

Data
Additional Information: clinicaltrials.gov (NCT04224987)
Data available: Yes
Data types: Deidentified participant data
How to access data: De-identified data and the corresponding data dictionary for this study are available through the Open Science Framework (https://osf.io/y5qgx/) at the time of publication.
When available: With publication

Supporting Documents
Document types: None

Additional Information
Who can access the data: Publicly available
Types of analyses: Publicly available
Mechanisms of data availability: Publicly available
